# Boosting Secondary Metabolite Production and Discovery through the Engineering of Novel Microbial Biosensors

**DOI:** 10.1155/2018/7021826

**Published:** 2018-07-09

**Authors:** Ulysses Amancio de Frias, Greicy Kelly Bonifacio Pereira, María-Eugenia Guazzaroni, Rafael Silva-Rocha

**Affiliations:** ^1^Medical School of Ribeirão Preto, University of São Paulo, Ribeirão Preto, SP, Brazil; ^2^Faculty of Philosophy, Science and Letters of Ribeirão Preto, University of São Paulo, Ribeirão Preto, SP, Brazil

## Abstract

Bacteria are a source of a large number of secondary metabolites with several biomedical and biotechnological applications. In recent years, there has been tremendous progress in the development of novel synthetic biology approaches both to increase the production rate of secondary metabolites of interest in native producers and to mine and reconstruct novel biosynthetic gene clusters in heterologous hosts. Here, we present the recent advances toward the engineering of novel microbial biosensors to detect the synthesis of secondary metabolites in bacteria and in the development of synthetic promoters and expression systems aiming at the construction of microbial cell factories for the production of these compounds. We place special focus on the potential of Gram-negative bacteria as a source of biosynthetic gene clusters and hosts for pathway assembly, on the construction and characterization of novel promoters for native hosts, and on the use of computer-aided design of novel pathways and expression systems for secondary metabolite production. Finally, we discuss some of the potentials and limitations of the approaches that are currently being developed and we highlight new directions that could be addressed in the field.

## 1. Background

Microorganisms have provided a variety of natural products (NPs) or secondary metabolites (SMs) with interesting chemical structures and bioactivities [1]. Typically, bacteria biosynthesize a range of distinct molecules, and many of them present remarkable biological activities acting as bioregulators, quorum-sensing/signaling molecules, and antimicrobial drugs [2]. However, the preponderance of NP molecules with clinical relevance was derived from Gram-positive bacteria, highlighting the* Streptomyces* genus [3]. Although many hundred thousand different NPs have been described (Dictionary of Natural Products 19.2, Copyright © 2011 Taylor & Francis Group), those molecules arising from four generic classes of biosynthetic systems have been reported most frequently. These are the (i) polyketides (PK), (ii) nonribosomal peptides (NRPs), (iii) isoprenoids, and (iv) shikimate derivatives [4–6]. The core structures biosynthesized by these four systems are generally defined as superimposed “scaffolds” whose “tailoring” modifications are implemented. These tailoring reactions modify the final structures of NPs by multiple mechanisms, including oxidation/reduction, macrocyclization, halogenation, glycosylation, acylation, phosphorylation, sulfation, methylation, and other chemical transformations [7].

While Gram-positive bacteria are without a doubt the most preeminent group for the isolation of SMs, it is important to notice that Gram-negative bacteria also represent an exciting group for biotechnological prospection. For instance, many clinically relevant NPs molecules produced by Gram-negative bacteria can be found in the Minimum Information about a Biosynthetic Gene cluster (MIBiG) database [8]. MIBiG implements robust and standard annotations for biosynthetic gene clusters (BGCs) and their molecules. NRPs and PKs are the most studied group of NPs in bacteria [9, 10]. Both form complex and diverse families of compounds with therapeutic applicability, such as molecules presenting cytotoxic/antitumor, antibacterial, antifungal, antimicrobial, cholesterol-lowering, immunosuppressant, and other activities. Briefly, NRPs and PKs are biosynthesized in modules by enzymes known as nonribosomal peptide synthetases and polyketide synthases, respectively [10]. Below, we present recent advances in synthetic biology approaches toward SMs production in bacteria.

## 2. Synthetic Biology Application for the Design of Novel Biosensors

**Figure 1:**
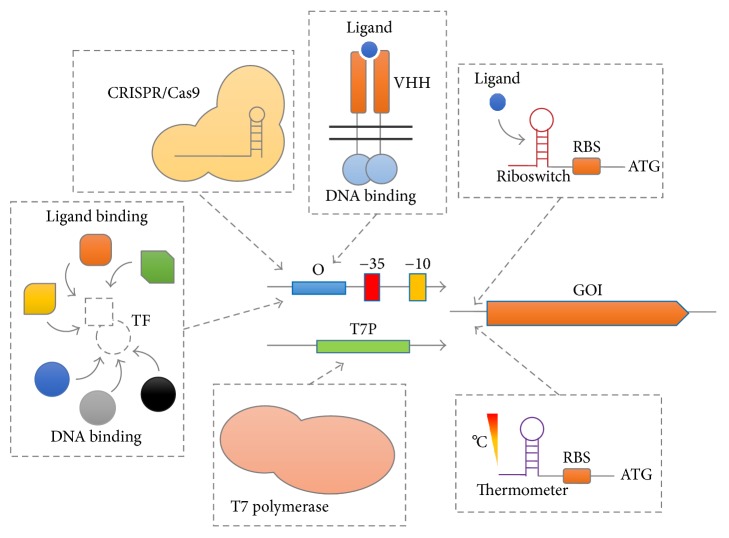
Main strategies to engineer novel control systems for SM production/discovery. As a central approach, several works have focused on the development of novel transcriptional control modules such as those based on the strong T7 RNA polymerase/T7 promoter. On a different perspective, natural or engineered small metabolite-responsive regulators have been used to control gene expression in response to a ligand of interest. More sophisticated approaches have focused on combining engineered ligand-binding and DNA-binding domains to create new expression devices. An entirely novel approach has been the usage of VHH antibody domains to couple ligand recognition to gene expression elements. Additionally, gene expression modulation using modified CRISPR/Cas9 modules is becoming more frequent every day. Finally, posttranslational regulation of protein production has been addressed through either the engineering of novel ligand-specific riboswitches or temperature responsive regulatory elements (thermometers). A full description of these main cases is presented in the text. GOI: gene of interest; O: operator, a* cis*-regulatory element; T7P: T7 promoter; RBS: ribosome binding site.

Recent years have been particularly productive for the generation of tools and approaches to enhance SM synthesis in bacteria. Under the umbrella of synthetic biology, many works have focused on the reconstruction of biosynthetic gene clusters in native or heterologous hosts or on the engineering of the regulatory network of the host itself, allowing an increasing expression of the genes of interest. These approaches have been extensively used for both Gram-positive and Gram-negative bacteria and have been expanded to nonmodel organisms through the generation of new genetic tools, as presented below. In terms of engineering or rewiring of the regulatory networks controlling biosynthetic gene clusters, there has been a tremendous effort to engineer new inducible systems in bacteria to use these elements either as control systems for gene pathway expression or as biosensors to monitor the production rate of the compounds of interest [11]. In this sense, Figure 1 represents schematically some of the main approaches developed recently, many of which are discussed below. The classical approach to enhance the production of a biosynthetic gene cluster is to place these elements under the control of a strong regulatory element such as the T7 RNA polymerase/T7 promoter system [12], which is highly used for large-scale protein production in* Escherichia coli*. Using this approach, Ross and coworkers captured a 34 kb gene cluster encoding the synthesis of an alterochromide lipopeptide from* Pseudoalteromonas piscicida* and expressed it under the control of a T7 expression system in* E. coli* [13]. This approach is particularly useful since the T7-based expression system allows an orthogonal expression device that can be introduced into a number of host strains [14], eliminating the necessity of redesigning native regulatory elements from the original host. Also, T7-based systems can be easily adapted for cell-free setups [15], such as the one generated for* in vitro* production of SM in* Streptomyces* [16]. Additionally, the use of strategies aiming at the assembly of artificial gene clusters could enhance even more the production of compounds of interest. This is the case of the AGOS (Artificial Gene Operon assembly System) presented by Basitta and coworkers (2017). This strategy allows not only the optimization of the pathway of interest, but also the introduction of gene diversity that could lead to the production of SM with different chemical modifications [17].

When the engineering of a particular host is intended, a frequent limitation is the lack of efficient genetic tools and induction systems essential for the success of the circuit of interest. In this sense, Phelan and coworkers (2017) have recently reported a set of new vectors for use in* Streptomyces venezuelae* aiming at the enhanced production of SMs [18]. By the same token, DeLorenzo and colleagues (2017) performed an extensive characterization of genetic parts related to fluorescent reporter systems, inducible systems, and biosensors for utilization in* Rhodococcus opacus* [19]. In that work, the authors were able to optimize inducible systems responsive to arabinose* (Pbad)*, anhydrotetracycline* (Ptet)*, and acetamide* (Pacet)*, while also combining genome mining and transcriptomic analysis to identify endogenous expression systems responsible for compounds such as phenol [19]. In fact, genome mining combined with transcriptomic analysis seems to be a very interesting way to find novel expression devices for use in synthetic biology projects in nonmodel organisms, and similar attempts have been reported for* Streptomyces* [20, 21]. In a very elegant report, Li and coworkers (2017) applied a statistical analysis to investigate the optimal condition for SM production in* Streptomyces coelicolor*. After finding the optimal conductions, the authors combined genome mining and transcriptomic profiles to identify native promoters with expression dynamics similar to those required to obtain the optimal SM production. By replacing the inducible system for the nearly identified native promoters, the authors generated stable strains producing enhanced amounts of the metabolites of interest [20]. Another example is presented by Khalid and collaborators (2017), who used a reassembled terpenoid-production pathway from* Streptomyces* initially placed under the control of the native regulator Fur22 and lately redesigned with native promoters from the primary metabolism of the bacterium, resulting in an optimized production strain [22]. These works thus demonstrate the power of combining approaches (statistical, induction, and mining) to enhance SM production in nonmodel organisms.

Once native promoters can be identified by a series of approaches, achieving the optimal production usually requires fine-tuning of the expression system used. This scenario makes the availability of sets of promoters with varying strengths imperative, allowing thus the construction of the circuits of interest. With this aim in mind, Yang and colleagues (2017) used promoter design together with random mutagenesis and selection to construct novel broad-host range promoters able to trigger gene expression in* E. coli*,* Bacillus subtilis*, and* Saccharomyces cerevisiae* [23]. This elegant work opened a new venue of possibilities to construct universal synthetic clusters that could be tested in several Gram-negative and Gram-positive hosts, and even across life kingdoms. While the work of Yang et al. provided a limited number of promoter variants analyzed by classical approaches, Rohlhill et al. (2017) used Fluorescence Activated Cell Sorting (FACS) coupled to next-generation sequencing (NGS) technologies to identify enhanced promoter variants generated by random mutagenesis of the formaldehyde-inducible promoter of* E. coli* [24]. This Sort-Seq technology could expand the existing capabilities to experimentally address novel promoters for SM-production engineering. Alternatively, novel computational approaches have been developed recently that allow* in silico* design of regulated promoters in* E. coli* [25, 26]. In combination with novel experimental approaches for the construction of combinatorial promoters with complex behaviors [27] and with models of transcriptional-factor/promoter interaction [28], these technologies could drastically simplify the way novel expression systems are engineered. However, a very drastic alternative for single-input promoter induction in bacteria has been the use of a mutated version of the CRISPR/Cas9 system not able to cleave DNA. In these systems, a nuclease-deficient Cas9 variant is used to block promoter activity by expressing a guide-RNA targeting RNAP binding site into the promoter, or by activating gene expression through the fusion of an RNAP subunit to Cas9 [29]. In fact, this strategy has been used to enhance the production of the lipodepsipeptide WAP-8294A in* Lysobacter enzymogenes* OH11 [30].

While tremendous progress in the identification/optimization of promoters has been achieved in the past years, there has also been an intensive search for novel transcriptional factors able to recognize new molecules of interest. As represented in Figure 1, these approaches have been based on (i) the design of biosensors by simply implementing an endogenous/heterologous regulator into a host of interest, (ii) the random or rational engineering of regulator variants to change the inducer specificity, or (iii) the reconstruction of novel transcriptional factors by fusing unrelated protein modules, generating new-to-nature, fully synthetic inducible systems. In the first case, Liu and coworkers (2017) used a Lys-type regulator named ShiR from* Corynebacterium glutamicum* to develop a shikimic acid biosensor in this organism. The use of this new biosensor in combination with FACS allowed optimizing the expression of the* tktA* gene, encoding a transketolase [11]. But while Liu et al. used a regulator able to recognize the compound of interest, there is currently a deficiency in the availability of regulators responsible for compounds of interest. In these cases, an alternative is to engineer by random mutagenesis or rational design novel regulators with improved response to a particular compound, and several examples have been reported over the years [31]. Of particular interest, Kasey and colleagues (2017) used the crystal structure of the TetR-type MphR regulator to select five candidate residues for saturation mutagenesis. Upon selection based on FACS, the authors were able to identify several regulator variants including a new pikromycin responsive transcriptional factor [32]. Again, while this approach allows the improvement in sensitivity toward suboptimal inducers, it still is restricted to compounds structurally similar to those recognized by the transcriptional factor of interest. In light of all the improvement on the engineering of synthetic regulators with specific ligand of DNA-binding selectivity, it is important to notice that the final sensitivity of the circuit is dependent not only on the sensitivity of the ligand-domain engineered, but also on the arrangement of DNA-binding sites existing in the target promoter [33]. Another radical approach is to construct novel transcriptional factors from the assembly of protein modules of interest. In this context, Younger and collaborators (2017) fused several zinc-finger DNA-binding modules to a maltose binding protein (MBP) domain to create novel synthetic regulators responsive to maltose [34]. In this context, this approach could be extended to other ligands of interest and would strongly facilitate the generation of novel inducible regulatory systems with different DNA- and ligand-binding specificities. In an even more drastic approach, Chang and colleagues (2017) fused the well-known DNA-binding domains (from LexA and CadC) to single-domain VHH (variable domains of camelid heavy chain only) antibodies to construct both cytosolic and transmembrane receptors for caffeine [35]. Once more, the potential of such technologies is tremendous as it allows the coupling of VHH domains (selected either naturally or from techniques such as phage display) to construct functional biosensors for molecules that could be internalized by the cell or that could be present only on the extracellular medium. While the progress on the engineering of novel transcriptional factors has been remarkable, it is important to notice that the single-cell behavior of the resultant expression system is often neglected. This is particularly important since many natural responsive systems can display a graded or bimodal expression behavior in single cells depending on the arrangement of the genetic circuit used [36].

Finally, another potential approach to construct tailored biosensors is to use natural or engineered trans-acting regulatory RNAs. In this sense, Jang et al. (2017) used SELEX (Systematic Evolution of Ligands by EXponential Enrichment) to construct novel riboswitches responsible for the flavonoid naringenin. This work is particularly important since the authors provided a defined aptamer design for optimal arrangement of the regulatory elements [37]. Using a different approach, Sen and coworkers (2017) used computational models of base pairing to construct temperature responsive trans-regulatory RNAs [38]. This type of regulatory element is particularly useful since many industrial conditions are prone to changes in temperature, and these elements could be used to provide fine-tuning for engineered pathways in bacteria. Yet, as the authors reported differences between the predicted and the experimental behaviors of the engineered structures, there is still room for improvements of the computational models used. In this sense, development of efficient* in silico* algorithms should permit creating functional RNA thermometers.

## 3. Metagenomics as a Source of Genetic Parts for SM Production in Bacteria

**Figure 2:**
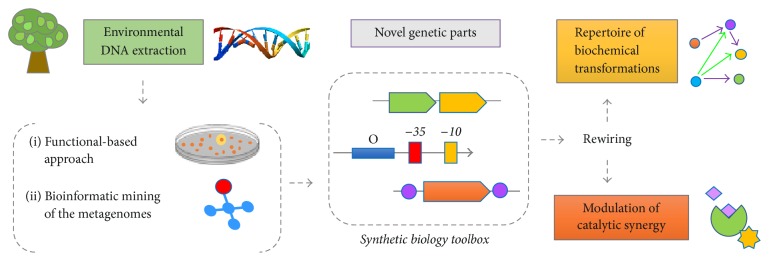
Metagenomics has become an important tool to improve and expand NP discovery from DNA retrieved from the microbial community inhabitants of environmental samples. Novel genetic parts (structural genes, regulators, terminators, peptide signals, transporters, etc.) are provided, which, when rewired, allow the creation of novel complex bioactive molecules.

Metagenomics allows searching in the huge biochemical array present in the genomes of microorganisms found in environmental samples, having access to most of the genetic material of bacteria that are recalcitrant to cultivation [39]. In this sense, novel functional gene clusters involved in the production of bioactive compounds can be identified by directly cloning or sequencing the DNA retrieved from the microbial community inhabitants of an ecosystem of interest (Figure 2) [40]. In the activity-based metagenomic approach, the construction and subsequent screening of metagenomic libraries allow identification of the targeted genes encoding the desired activities [41]. Thus, large-insert libraries, usually constructed in cosmids, fosmids, or BACs (bacterial artificial chromosomes), would allow the recovery of complete biosynthetic pathways or the functional expression of large multienzyme clusters (as in the case of nonribosomal peptide synthetases (NRPS), polyketide synthases (PKS), and terpene synthases, to name a few) [42]. Normally, PKS type I and NRPS's pathways are organized in large assembly operons going from 20 kb to 100 kb in length [43]. Accordingly, several examples in the literature show that metagenomics has been successfully applied for the identification of novel pathways coding for bioactive compounds in diverse environments [44–49]. Although the functional-based approach allows obtaining completely original genes in an independent-sequence way, numerous studies using next-generation sequencing techniques and subsequent bioinformatic mining of the metagenomes have also achieved novel gene clusters involved in secondary metabolites production [50–53].

In this sense, metagenomics has become a proper methodological tool to improve and expand NPs discovery from natural sources, contributing with novel genetic parts (such as structural genes and regulatory sequences), acting as an important contributor to the expansion of the synthetic biology toolbox (Figure 2) [54, 55]. Secondary metabolite genes are organized in clusters or operons leading to a well-ordered biosynthesis of molecules in multiple sequential steps by a set of functionally interconnected enzymes [56]. Novel combinations and rewiring of these enzymes performing a huge repertoire of biochemical transformations, along with proper modulation of catalytic synergy, would permit the design and generation of innovative complex bioactive molecules. For instance, Smanski and coauthors (2014) developed an approach exploiting the modularity of a refactored* Klebsiella oxytoca* nitrogen fixation gene cluster that led to the functional optimization of the operon by combinatorial design and assembly of 103 biological parts [57]. Also, a study reported a plug-and-play pathway refactoring workflow using expression cassettes for high-throughput pathway construction in* E. coli* and* S. cerevisiae* [58]. As a proof of principle, a total of 96 pathways for combinatorial carotenoid biosynthesis were built successfully [58]. In an elegant study, Freestone and collaborators (2017) discovered a novel phosphonoacetic acid by pathway refactoring of a gene cluster from* Streptomyces* sp. strain NRRL F-525 using for expression the production host* S. lividans *[59]. In parallel, efforts are conducted for the optimization of biosynthesis of compounds through the creation of algorithms considering a plethora of genetic and nongenetic factors [60, 61].

On the other hand, a current significant limitation in recovery of natural products from metagenomes is the use of* E. coli* as a host, a Gram-negative bacterium that is distant phylogenetically from microbes that are native producers of NPs [62]. This way, the strongest producers of diverse secondary metabolites broadly used in several therapeutic applications are the Gram-positive bacteria belonging to the phylum Actinobacteria [63–65]. Restrictions related to codon usage, transporters, regulatory signals, proper folding of proteins, and cofactor availability, as well as limited precursor availability for secondary metabolite synthesis, are some of the main constraints that restrict* E. coli* usage as a host for NPs discovery in metagenomic libraries [66]. However, a considerable number of efforts involving systems and synthetic biology approaches along with metabolic engineering are already in progress to transform Actinobacteria members in sophisticated heterologous expression platforms for NP biosynthesis. For instance, different strains of* S. coelicolor* have been genetically engineered to enhance secondary metabolite expression, involving deletions in endogenous gene clusters [67], removal of transcriptional factors [68], or modifications of native promoters [69]. Moreover, systematic minimization of the genome of the industrial microorganism* Streptomyces avermitilis* was carried out to remove nonessential genes, leading to the creation of a versatile model host for heterologous expression of NPs [70]. In addition, different genetic tools for genetic manipulation and genome editing (i.e., the prominent CRISPR/Cas system) were already developed in Actinobacteria [69, 71–76]. In conclusion, while there are still several limitations that need to be overcome to optimize these bacteria as effective host strains for the screening of metagenome libraries, the work at present including synthetic biology approaches for chassis engineering and molecular tools development seems to be certainly promising.

## 4. Potential of NPs Originating from Gram-Negative Bacteria

**Figure 3:**
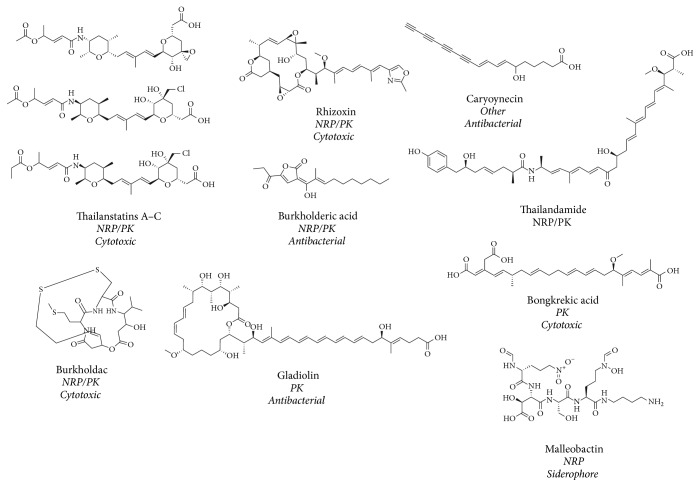
Chemical diversity of NPs identified from* Burkholderia.* The main groups of molecules belonging to NRP and PK are shown.

As stated before, while Gram-positive bacteria have the highest potential for SM production, this topic has been extensively reviewed elsewhere [77]. Additionally, one of the main advantages of using Gram-negative bacteria in comparison to Gram-positive is the abundant portfolio of genetic tools currently available, especially those for the metabolically versatile bacterium* Pseudomonas putida* [78]. Therefore, we focus here on the remarkable potential of biosynthetic gene clusters found in Gram-negative bacteria and on the metabolic engineering of SM production in these organisms. In this sense, the* Burkholderia* genus has a powerful enzymatic machinery for the biosynthesis of many distinct natural products with clinical interest. Furthermore, after Actinobacteria,* Burkholderia* presents the second highest percentage of clusters related to the biosynthesis of PKS and NRPS molecules [79]. The genus* Burkholderia* is cosmopolitan and has been isolated from the most diverse types of environments, from soil and water to the human lung [80]. This genus comprises more than 90 species, according to the German Collection of Microorganisms and Cell Cultures (DSMZ) database. Considering its potential for NPs biosynthesis, several studies have demonstrated that the* Burkholderia* genera are able to produce important molecules such as rhizoxin [81], bongkrekic acid [82], thailanstatin [83], burkholdac [84], spliceostatin [85], thailandamide [86], bactobolin [87], and gladiolin [88] (Figure 3). Two interesting examples of NPs isolated from* Burkholderia* are burkholdacs and gladiolin. The isolation of the bicyclic depsipeptide burkholdacs A and B was reported by Biggins (2011) as histone deacetylase (HDAC) inhibitors. The burkholdacs were identified as a hybrid NRP/PK; these molecules were obtained through overexpression of a transcription factor associated with gene clusters in* B. thailandensis* E264 [84]. Recently, Song et al. (2017) [88] reported the identification of gladiolin from* B. gladioli*, a compound molecularly comparable to the unstable antibiotic etnangien [89, 90]. Like etnangien, gladiolin inhibits RNA polymerase, providing an antibacterial activity against* Mycobacterium tuberculosis*. Moreover, purified gladiolin was tested against ESKAPE panel of pathogens and showed also a moderate activity against methicillin-resistant* Staphylococcus aureus* (MRSA) [88]. In addition to* Burkholderia*,* Pseudomonas* is also a cosmopolitan microorganism and possesses the ability to biosynthesize a wide range of specific metabolites, emphasizing the production of NRP. Many of these NRPs showed significant biological activities; the following antimicrobials can be cited as examples: cichofactin [91], tolaasin [92–95], sessilin [96, 97], kalimantacin [98–100], obafluorin [101, 102], arthrofactin [103], xantholysin [104], poaeamide [105, 106], and WLIP [107–110]. In this sense, while Gram-negative bacteria have been primarily used as hosts for the assembly of novel biosynthetic pathways, still there is a tremendous potential for the screening and production of novel bioactive molecules from these organisms.

## 5. Metabolic Engineering of Gram-Negative Bacteria for SM Production

Over the past years, several groups have worked on the metabolic engineering of SM production in bacteria. In this sense,* E. coli* is one of the most characterized Gram-negative bacteria and many strategies have been developed for the genetic manipulation and engineering of this organism. Since the success of the first ever genetically modified* E. coli* in 1973, this bacterium became the pioneer in the field of genetic engineering [111]. One interesting example is the use of cocultures of* E. coli* for the producing of flavan-3-ols, a subclass of flavonoid molecules, which have broad pharmaceutical applications. This approach produced a 970-fold improvement when compared to previous attempts, and it allowed the optimization of diverse factors such as carbon source, induction temperature, induction point, inoculation ratio, and strain choice [112]. Also, the heterologous expression of carotenoid genes from* Pantoea ananatis *in* E. coli* produced great amounts of zeaxanthin [113], a carotenoid synthesized by some plants, bacteria, and fungi [114], used against age-related macular degeneration and also in the food industry [115, 116]. For this, the authors used the tunable intergenic regions approach to coordinate the expression of the* crtY *and* crtZ *genes [113]. Yet, genes related to the production of myrcene, an acyclic monoterpene, are also being customized in* E. coli* strains [117]. Myrcene, a monoterpene compound, has been considered as a starting material for the synthesis of more complex compounds, and it is utilized in flavors, fragrances, cosmetics, vitamins, and pharmaceuticals [118]. Through the heterologous expression of the mevalonate (MVA) pathway, the level of myrcene increased 34 times (58.19 ± 12.13 mg/L) [117]. Additionally, recently, the US Department of Energy has listed the glutamate derivate 2-pyrrolidone as an important C4 “Top Value-Added Chemical from Biomass” [119]. 2-Pyrrolidone is a high-value product considering its great commercial applicability. It can be used in ring-opening polymerization to produce nylon-4, an enhanced nylon type fiber with more thermal stability and hydrophilicity than its precursors. With that in mind, a group from California has engineered a recombinant* E. coli* strain capable of producing 2-pyrrolidone using glutamate as a substrate [120]. To achieve that, two ORFs of the type I PK gene clusters were first identified by* in silico* analysis, both predicted to be AMP-dependent synthetases. Recombinant* E. coli* expressing a glutamate decarboxylase and one of the synthetases showed an improvement in the 2-pyrrolidone production, with an efficiency of 25% [121]. Still, a coexpression method to produce* trans*-resveratrol in* E. coli* was put forward by Wang et al. (2017) [122]. Resveratrol is a secondary metabolite, member of the stilbene family found in a wide range of plant sources [123], aromatherapy products, and dietary supplements. Stilbenes are being used in humans for the prevention of cancer, heart diseases, and neurodegenerative diseases [123].

In addition to* E. coli*,* P. putida* has also been quite used in metabolic engineering.* P. putida* is a Gram-negative bacterium that metabolizes a wide range of natural and synthetic organic compounds, and this competence prompted many studies to use* P. putida* as a biocatalytic agent in the industrial and environmental area [124, 125]. Considering that, Simm et al. (2016) published a study in which they transformed* P. putida* with two genes from* E. coli*, encoding active GGDEF and EAL domains, which are related to c-di-GMP production and degradation, respectively [126]. The* P. putida* mutants were able to control biofilm formation according to specific catalytic needs, for example, for biodegradation of the environmental pollutant 1-chlorobutane. The authors showed that, by adding cyclohexanone to the culture medium, the secondary metabolite derived from haloalkane increased twice in the* P. putida* biofilm forming cells when compared to the planktonic cells forms [127].

## 6. Future Perspectives

As presented here, bacteria from several groups have the gene clusters for the production of secondary metabolites with potential applications in biomedicine. With the advance of postgenomic tools, there has been tremendous progress in the development of novel technologies (i) to engineer natural produces of bioactive compounds to increase productivity, (ii) to reassemble novel biosynthetic pathways into native or heterologous hosts, (iii) to mine novel clusters from metagenomes, and (iv) to monitor/discover novel metabolites using biosensors. It is important to notice that, with the advent of synthetic biology, a number of computational tools and approaches have emerged, which allowed the design of novel regulatory elements and pathways for SM production. It is reasonable to think that using strong computational tools more and more should become routine in the field, and this should have a remarkable impact in the area by allowing a more accurate design or novel pathways, speeding up the process of SM discovery. Additionally, novel genetic tools are continually being produced, and their utilization in bacterial species lacking an efficient genetic toolbox should allow the exploitation of novel microorganisms and develop their full potential. By the same token, metagenomic approaches hold potential to provide novel genetic parts either for the reprogramming of bacteria for SM production or as modules for the assembly of synthetic gene clusters, and these methodologies should be further addressed in the future. Finally, all the aforementioned improvements once coupled with high-throughput screening and engineering protocols should permit a strong increase in the SM discovery rate, allowing the identification of novel bioactive molecules.
